# Correlation between Peri-Implant Marginal Bone Loss Progression and Peri-Implant Sulcular Fluid Levels of Metalloproteinase-8

**DOI:** 10.3390/jpm12010058

**Published:** 2022-01-06

**Authors:** Renzo Guarnieri, Alessio Zanza, Maurilio D’Angelo, Dario Di Nardo, Andrea Del Giudice, Alessandro Mazzoni, Rodolfo Reda, Luca Testarelli

**Affiliations:** 1 Department of Oral and Maxillofacial Sciences, Sapienza University of Rome, 00161 Rome, Italy; renzoguarnieri@gmail.com (R.G.); alessio.zanza@uniroma1.it (A.Z.); Maurilio.dangelo@uniroma1.it (M.D.); andrea.delgiudice@uniroma1.it (A.D.G.); alessandro.mazzoni@uniroma1.it (A.M.); rodolfo.reda@uniroma1.it (R.R.); luca.testarelli@uniroma1.it (L.T.); 2 Private Periodontal Implant Practice, 31100 Treviso, Italy

**Keywords:** dental implant, marginal bone loss, metalloproteinase-8, sulcular fluid

## Abstract

Objectives: The aim of this retrospective study was to analyze peri-implant marginal bone loss levels/rates and peri-implant sulcular fluid levels/rates of metalloproteinase-8 in three timeframes (6 months post-surgery—restoration delivery (T0)—and 6 (T6) and 24 (T24)-months post-loading) and to evaluate if there is a correlation between peri-implant sulcular fluid levels of metalloproteinase-8 and peri-implant marginal bone loss progression. Materials and Methods: Two cohorts of patients undergoing implant surgery between January 2017 and January 2019 were selected in this retrospective study. A total of 39 patients received 39 implants with a laser-microtextured collar surface, and 41 subjects received 41 implants with a machined/smooth surface. For each patient, periapical radiographs and a software package were used to measure marginal bone loss rates. Implant fluid samples were analyzed by an enzyme-linked immunosorbent assay (ELISA) test. The modified plaque index, probing depth, and bleeding on probing were also recorded. Results: High marginal bone rates at T24 were strongly associated with elevated rates between T0 and T6. The levels of metalloproteinase-8 were significantly more elevated around implants with marginal bone loss, in relation to implants without marginal bone loss. Marginal bone loss (MBL) rates at 24 months were associated with initial bone loss rates and initial levels of metalloproteinase-8. Conclusions: Peri-implant marginal bone loss progression is statistically correlated to peri-implant sulcular fluid levels of metalloproteinase-8. Moreover, the initial high levels of marginal bone loss and metalloproteinase-8 can be considered as indicators of the subsequent progression of peri-implant MBL: implants with increased marginal bone loss rates and metalloproteinase-8 levels at 6 months after loading are likely to achieve additional marginal bone loss values.

## 1. Introduction

Peri-implant health or/and inflammatory conditions were defined at the 2017 World Workshop on the Classification of Periodontal and Peri-implant Diseases and Conditions [1] as appearing in four forms: (1) peri-implant health, (2) peri-implant mucositis, (3) peri-implantitis and (4) hard- and soft-tissue deficiencies. However, some questions remain controversial; namely, in which condition among those described is an implant with marginal bone loss (MBL) to be considered, and what are the acceptable levels of MBL in order to establish a healthy or diseased peri-implant condition [2,3]. MBL is influenced by numerous variables related to surgical trauma, implant design, the general health of the patients, bone substratum, patient habits, the implant–abutment connection, prosthetic considerations, etc., [4,5], but some key questions remain unanswered. Not only does the need to define the etiology of MBL remain, but also to distinguish between physiological and pathological peri-implant bone losses. Recent studies [6,7], highlighting that MBL rates rather than raw MBL data might improve the ability of clinicians to predict a change in health status of peri-implant tissues, offered some important clues on this issue. However, to date, it still seems impossible to generalize a parameter or a measurement that allows one to define a physiological or pathological MBL without an evaluation of the specific characteristics of each single implant/patient, with each playing an important role in the prognosis [6,7].

In dentistry, the conventional diagnostic tools for peri-implant tissue inflammatory status are clinical and radiographic examination [8]. These can provide information about the extent of peri-implant tissue loss/destruction but cannot at present predict the risk of the inflammation progression [9]. Various host-derived biochemical markers have been suggested in relation to the diagnosis of peri-implant inflammatory diseases and to monitoring their course and treatment [10,11,12]. These include peri-implant sulcular fluid (PISF), salivary and mouth rinse analysis of inflammation-associated molecules and tissue-destruction markers, various enzymes and other proinflammatory mediators originating from supporting peri-implant tissues and inflammatory cells. Among enzymes, an important diagnostic role is attributed to metalloproteinases (MMPs). MMPs are endopeptidases that form a gene family of at least 25 genetically distinct but structurally related zinc-dependent proteinases that are synthesized in the latent pro-form and activated in the extracellular space or at the cell surfaces [13,14]. Substrates for these enzymes are collagen types I, II, III, and IV; accordingly, they are involved in the degradation of extracellular matrix proteins such as laminin, collagens, proteoglycans, and fibronectin, which lead to increased migration of inflammatory cells and destruction of the tissue structure. In addition, MMPs can process or activate host defense molecules and pro-inflammatory mediators, as well as modulate various cellular signaling pathways [15,16]. MMPs, together with the factors associated with their regulation, are highly implicated in various inflammatory tissue destructive pathologic conditions, such as periodontitis [15,16,17,18], caries [19,20], lung diseases [21] rheumatoid arthritis and osteoarthritis [22]. MMPs are also involved in physiological remodeling [23], wound healing [24], tooth development and eruption [25] and embryonic morphogenesis [26]. MMP-8 (collagenase-2) is the major tissue-destructive MMP in the periodontitis-affected gingiva [15,16,17,18], and the elevated MMP-8 levels have also been demonstrated in the peri-implantitis sites [27,28,29]. Moreover, several studies assessing PISF MPP-8 levels in different peri-implantitis lesions reported a positive correlation between their concentration and clinical inflammatory conditions around implants [28,29,30]. Nowadays, it is believed that the measurement of the concentration of MMP-8 in the peri-implant sulcular fluid (PISF) may be very helpful in assessing the degree of inflammation within peri-implant tissues, particularly in the early inflammation phase [29,31,32,33,34,35,36]. Its advantage, beyond traditional diagnostic procedures (e.g., bleeding on probing, pocket depth, radiographic examination), is enhanced diagnostic sensitivity and specificity, including the ability to predictively detect health or/and inflammatory status before clinical and radiographic measurements indicate pathologic changes [32,33,34,35,36]. 

The present study aimed:

(1) to determine a cutoff point for discriminating between low and high bone loss-type (BLT) implants, considering a threshold of 2 mm at 24 months [6];
(2) to evaluate a possible correlation between peri-implant marginal bone loss progression and peri-implant sulcular fluid levels of aMMP-8; and
(3) to examine the patient and clinical variables that might influence MBL progression.

## 2. Materials and Methods

The following study was conducted in accordance with the Helsinki Declaration and following the approval of the ethics committee of the Department of Oral and Maxillofacial Sciences of “La Sapienza” University of Rome (prot. N. 528/17).

All subjects were consecutively enrolled in a private practice office. Inclusion criteria included:

- age > 18 years,
- presence of at least one edentulous site in posterior areas,
- physical status of I or II according to the American Society of Anesthesiologists (ASA) classification system, absence of systemic diseases or conditions known to alter bone metabolism,
- presence of stable periodontal condition (patient had to present at the first visit an inter-proximal attachment loss < 3 mm and/or radiographic bone loss < 30% of root length in <30% of sites) [37].

Two groups of patients, treated with two different kinds of dental implants were enrolled between January 2017 and January 2019. Group 1, comprising 41 patients, received 41 Tapered Internal TRX implants (BioHorizons, Birmingham, AL, USA). Group 2, comprising 39 patients, received 39 Tapered Internal TLX (BioHorizons, AL, USA). TRX and TLX implants have the same tapered macro design and the same body grit-blasted surface; TRX implants have a maximum coronal of 0.3 mm on the collar machined (M) surface, while TLX implants have a maximum coronal of 1.8 mm on the collar laser-microgrooved (LM) surface (Appendix A).

Surgical and restorative procedures: All surgical procedures were conducted under local anesthesia with Articaine Hydrochloride/Adrenaline Hydrochloride 1/100,000 (Ultracain, Aventis Inc., Frankfurt, Germany) by the same surgeons (RG and LT) in accordance with the manufacturer’s recommendations. Each patient received antibiotic medication with amoxicillin/clavulanic acid tablets (875/125 mg, TID immediately after surgery and for 7 days) or, if allergic to penicillin, clindamycin tablets (300 mg, TID immediately after surgery and for 7 days), and anti-inflammatory medication (ibuprofen 600 mg, every 4–6 h as needed to a maximum of 3600 mg/day). Suture removal was performed 2 weeks post-surgery. Same healing abutments (Standard Healing Abutment, BioHorizons, AL, USA) in both groups were placed in a second surgical procedure after 5 months of healing, and implant-supported prostheses were delivered 4 weeks later. TRX and TLX implants have the same internal connection, and received the same prosthetic UCLA type abutments (BioHorizons, AL, USA) to connect implants with the screwed restoration. All definitive restorations were single crowns. 

Radiographic evaluation: The radiographs were taken at implantation surgery (baseline = BSL), at the final restoration delivery (T0 = implant loading), and at 6 (T6) and 24 (T24) months after functional loading. Endoral radiographs were performed with a paralleling technique using a Rinn film holder with a rigid film-object X-ray source. For the radiograph procedure, a silicone index material was fixated to the residual dentition, and a radiograph holder was constructed for each patient. This technique ensured that the same position of the radiograph film could be reproduced at each visit, and the angle of the radiograph would not deviate. The radiographs were taken in high-resolution mode (Vista Scan Durr Dental, Durr Dental Italy S.r.l, Muggiò, Italy) with a dental X-ray machine (TM 2002 Planmeca Proline CC, Planmeca Group Helsinki, Helsinki, Finland) equipped with a long tube that operated at 70 Kw/7.5 mA. Specialized software (DBSWIN software, Durr Dental Italy S.r.l, Muggiò, Italy) was used for linear measurements of marginal bone changes. The following radiographic measurements were performed:

- radiographic implant length (IL): distance (in mm) between the implant coronal margin and the implant apex, as assessed at the midportion of the implant; and
- residual bone height at the mesial (MI) and distal (DI) aspects of the implant: distance (in mm) between the line linking the coronal implant margin, and the first contact of the crestal bone on both mesial and distal sides of the implant.

To account for radiographic distortion, radiographic measurements on each radiograph were adjusted for a coefficient derived from the following ratio: true length of the implant/IL. 

The radiographic marginal bone loss (MBL) was calculated by subtracting the marginal bone level at BSL from the marginal bone level at T0, T6 and T24. For each implant, the mean MBL was calculated by adding the MI to the DI and dividing the result by two. 

All measurements were carried out by a single trained examiner (RG) who had previously undergone a calibration session for radiographic assessment on a sample of 5 patients treated with the same implant system and not included in this study. To ensure reproducibility, radiographic measurements of the same 5 TRX and 5 TLX implants were repeated after 7 days, assessing an intra-examination reliability of 90%. 

Clinical examination: At T0, T6, and T24, at each implant site, the following clinical parameters were assessed: modified plaque index (mPI), modified gingival index (mGI), bleeding on probing (BoP), and probing depth (PD) [8]. mPI, mGI, BoP, and PD measurements were performed at 6 aspects per implant. mPI was scored as: 0 = no detection of plaque; 1 = plaque only recognized by running a probe across the smooth marginal surface of the implant; 2 = plaque can be seen by the naked eye; 3 = abundance of soft matter. mGI was scored as: 0 = no bleeding when a periodontal probe is passed along the mucosal margin adjacent to the implant; 1 = isolated bleeding spots visible; 2 = blood forms a confluent red line on mucosal margin; 3 = heavy or profuse bleeding. BoP was scored as 0 = absence; 1 = presence; PD was measured in mm. In addition, at T0, T6 and T24, full mouth plaque score (FMPS) and full mouth bleeding score (FMBS) were assessed.

Additional recorded data included: age, gender, smoking, and alcohol consumption at study enrolment. Patients were classified as smokers (if he/she smoked at least 1 cigarette/day) or non-smokers and were considered as alcohol consumers if their intake was >10 g/day (calculated as mL drunk × alcohol content of the drink × 0.79 (alcohol density). 

Peri-implant sulcus fluid (PISF) sample collection: At T0, T6 and T24, PISF samples were analyzed for the MMP-8 concentration. The sampling site was prepared by means of the removal of excess saliva with a short, gentle blast of air. A sterile PISF collection strip was placed apically as deeply as possible into the sulcus at the sampling site using tweezers. aMMP-8 levels were determined by the aMMP-8 PoC/chairside mouth rinse test, (PerioSafe^®^), in combination with a digital reader, (ORALyzer^®^), following the manufacturer’s instructions [32,38]. This test is based on a lateral flow sandwich immunoassay (DIPSTICK test) using the highly specific monoclonal antibodies MoAB 8706 and MoAB 8708, conjugated to latex particles. The ImplantSafe^®^ test has a high sensitivity (90%) and specificity (70–85%) [32].

### Statistical Analysis

Implants with low BLT and high BLT were classified using a nonparametric receiver operating curve (ROC) according to their MBL value and aMMP-8 concentration. MBL and aMMP-8 rates were calculated at 6 months post-surgery (T0 = restoration delivery) and at 6 (T6) and 24 (T24) months post-loading by dividing the MBL and aMMP-8 level at the restoration delivery interval (6 months post-surgery) by the number of months elapsed between the implant placement and implant loading stages. Three MBL rates (T0r, T6r, and T24r) were computed in millimeters/month (mm/m), and three aMMP-8 rates (T0r, T6r, and T24r) were computed in nanograms/milliliters/months (ng/mL/m). MBL- and aMMP-8 T0r, T6r, and T24r were analyzed by a linear mixed model using the patients as clusters and the implant as a unit of analysis by dividing the MBL and aMMP-8 level at the restoration delivery interval (6 months post-surgery) by the number of months elapsed between the implant placement and implant loading stages. Age, gender, smoking habits, alcohol intake, mPI, mGi, PD, BoP, location, implant length, implant diameter, and implant collar surface were considered as variables. To test the association between T0, T6, and T24 cutoffs, a crosstabs procedure for complex samples was applied. A 0.005 significance level per comparison, and the Bonferroni correction to take into account the large number of potential predictors, were used.

## 3. Results

**Figure 1:**
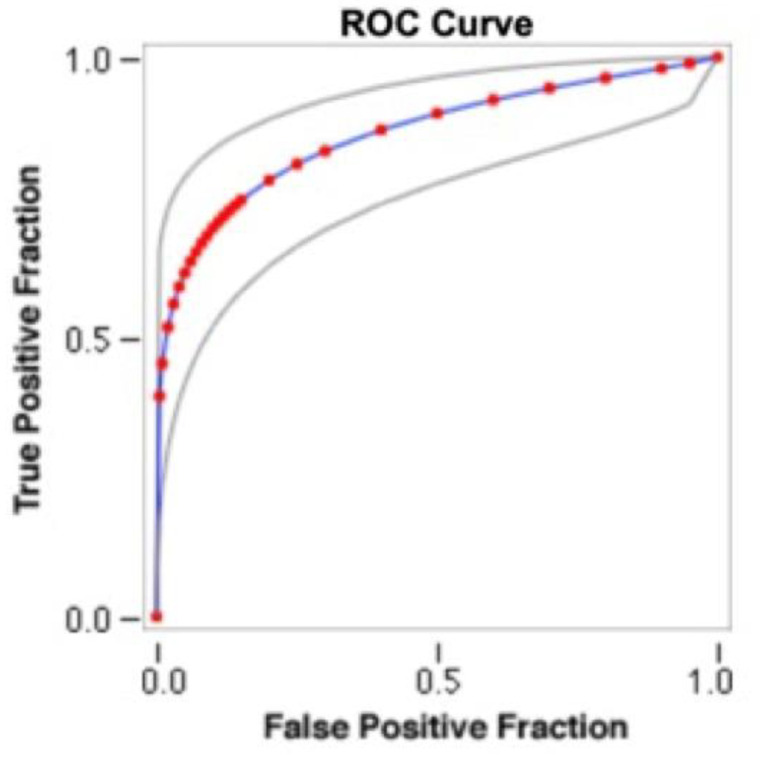
Nonparametric receiver operating curve (ROC) analysis for MBL (mesial + distal/2) at 24 months. The areas under the ROC (AUC) are significant.

Table 1 and Table 2 display the socio-demographic and clinical features of the study sample. None of the included implants were diagnosed with peri-implantitis (1–3), which prevents any extrapolation of findings to implants with a substantial disease. Implants with a LM vs. M collar surface, at T0, T26, and T24 presented a mean MBL of 0.469 ± 0.2 mm vs. 0.732 ± 0.4 mm, 0.512 ± 0.3 mm vs. 1.424 ± 0.7 mm, and 0.63 ± 0.26 mm vs. 1.674 ± 0.9 mm, respectively. At each follow-up examination, a statistically significant difference was noted between the two groups: 

Marginal Bone Loss: Receiver operating curve analysis (Figure 1) for MBL indicated that the optimal cutoff value for categorizing implants as high BLT or low BLT was 1.501 ± 0.33 mm, according to the kappa index. Therefore, 35 (43.5%) implants were classified as high HLT and 45 (56.5%) as low BLT. 

**Figure 2:**
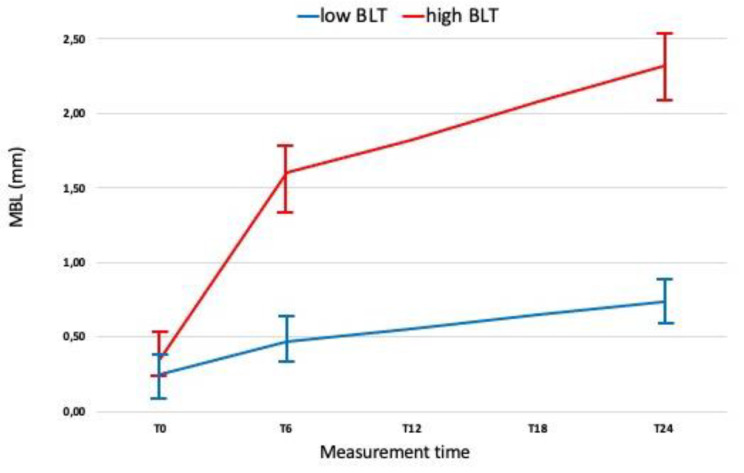
Marginal bone loss values as a function of time. Healing time (T0 = 6 months from surgery—restoration delivery); T6 and T24 were measured at 6 and 24 months after functional loading.

Marginal bone loss values as a function of time are indicated in Figure 2.

**Figure 3:**
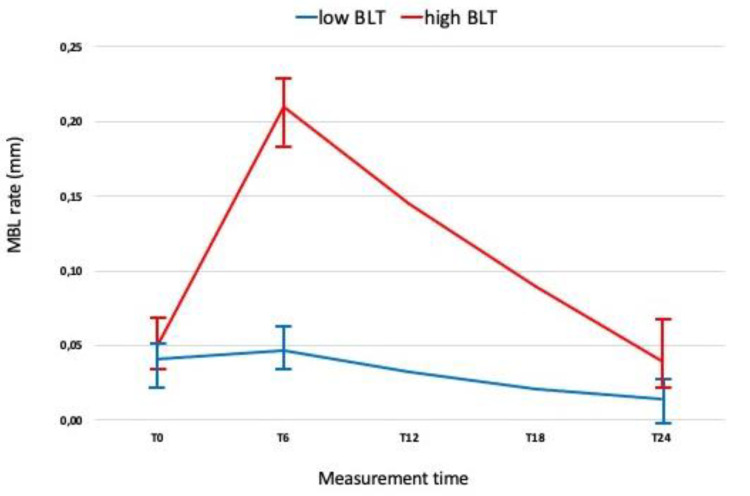
Marginal bone loss rates as a function of time. Healing time (T0 = 6 months from surgery—restoration delivery); T6 and T24 were measured at 6 and 24 months after functional loading.

**Figure 4:**
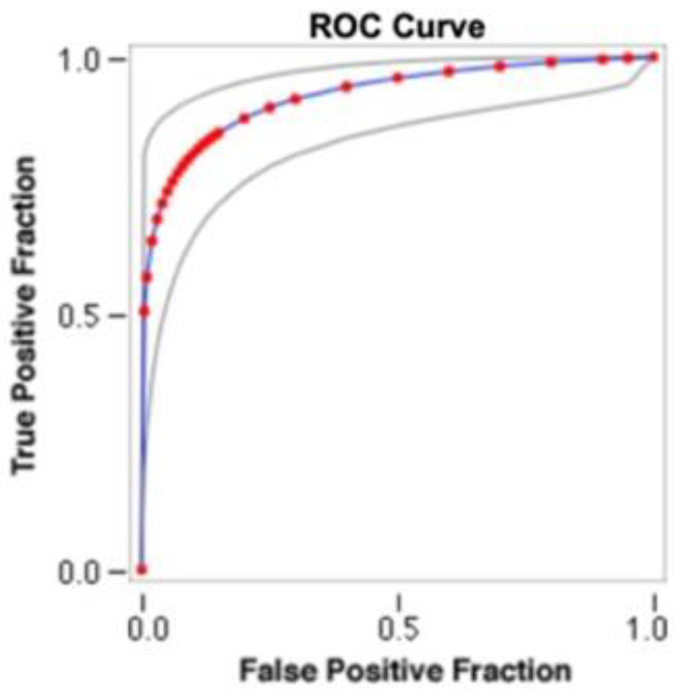
Nonparametric receiver operating curve (ROC) analysis for aMMP8 levels at 24 months. The areas under the ROC (AUC) are significant.

Figure 3 and Figure 4 show the time course of absolute MBL values and MBL rates as a function of the time since the interventions. The absolute MBL value increased as a function of time, whereas the MBL rate increased up to 6 months after the intervention. Table 3 reports the descriptive statistics for the three rates as a function of the main study factors. 

The mixed linear model analysis yielded significant effects for BLT (*p* < 0.001), implant collar surface (*p* < 0.001), time (*p* < 0.001), smoking (*p* < 0.001), and the interaction of BLT with time (*p* < 0.001). 

Analysis of the BLT–time interaction showed that the implant collar surface (*p* < 0.001) and time (*p* < 0.001) were significant for the low BLT, with a lower MBL rate for the LM 0.033 mm/m SD 0.01) vs. M (0.056 mm/m, SD 0.02) surface. The Bonferroni-corrected comparison between times indicated that the rate was lower for T1 (0.031 mm/m SD 0.01) than for T2 (0.047 mm/m SD 0.02) or T3 (0.041 mm/m SD 0.02), which showed no difference between them. The implant collar surface (*p* < 0.001), smoking (*p* = 0.001), and time (*p* < 0.001) were significant for high BLT, again observing higher MBL rates for the M (0.069 mm/m SD 0.02) vs. LM surface (0.084 mm/m SD 0.03). 

T0 rates were affected by the implant collar surface (*p* < 0.001), smoking (*p* < 0.001) and age (*p* < 0.001). T6 rates were influenced by the implant collar surface (*p* < 0.001), PD (*p* < 0.001), BoP (*p* < 0.001), and smoking (*p* < 0.001). T24 rates were influenced by the implant collar surface (*p* < 0.001), PD (*p* < 0.001), BoP (*p* < 0.001), and smoking (*p* < 0.001). 

Crosstab statistics for complex samples indicated that MBL values at T0 and T6 were significantly associated with those at T24, adjusted F = 37.36 (OR = 2.41) and adjusted F = 184.07 (OR = 9.14), respectively (*p* < 0.001); 81% of low BLT implants at T3 were low BLT at T2, while 86% of high BLT implants at T3 were also high BLT at T2. Moreover, 84% of low BLT implants at T3 were low BLT at T1, whereas 46.3% of high BLT at T3 were also high BLT at T1. 

Mixed linear model analysis showed that differences between T6 and T0 rates were explained by the implant collar surface (*p* < 0.001), smoking (*p* < 0.001), and age (*p* = 0.044), whereas differences between T24 and T6 rates were accounted for by the implant collar surface (*p* < 0.001) and BoP (*p* < 0.001). 

Complex samples crosstab indicated that implants with an MBL rate > 0.0765 mm/m SD 0.02 (0.38 mm/m SD 0.01 MBL at T0) at T6 were much more likely to have an MBL of ≥2 mm at T24 (OR = 9.39, 95% CI [6.67 13.24]). Thus, 81% of implants with MBL of <2 mm at T24 had an MBL rate ≤ 0.0765 mm/m at T6, whereas 84% of those with MBL of ≥2 mm MBL at T24 had a rate above this T6 cutoff value.

**Figure 5:**
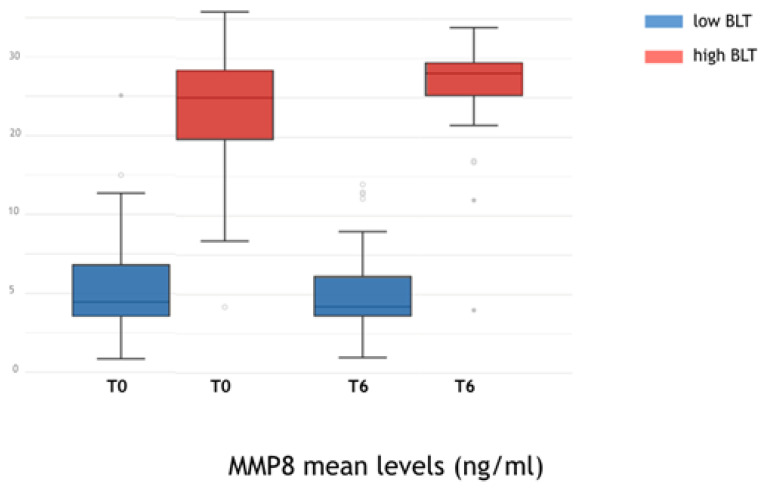
Mean MMP-8 levels recorded around low BLT and high BLT implants at T0 (restoration delivery) and T6 (6 months after functional loading).

**Figure 6:**
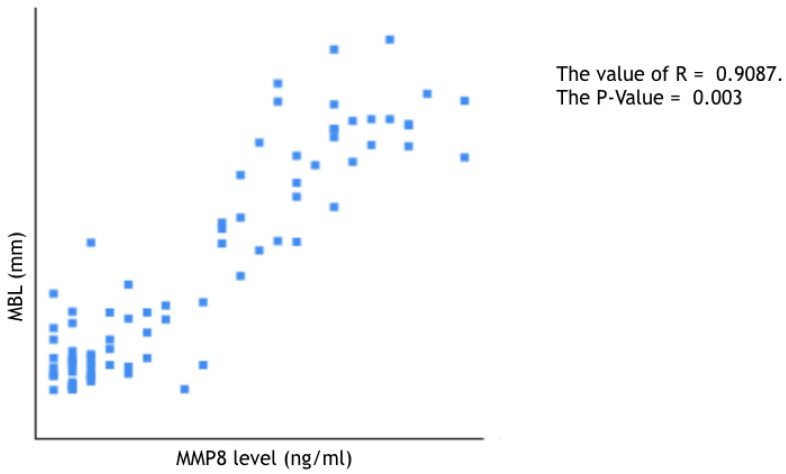
Pearson correlation between MBL and MMP-8 concentration at T6.

**Figure 7:**
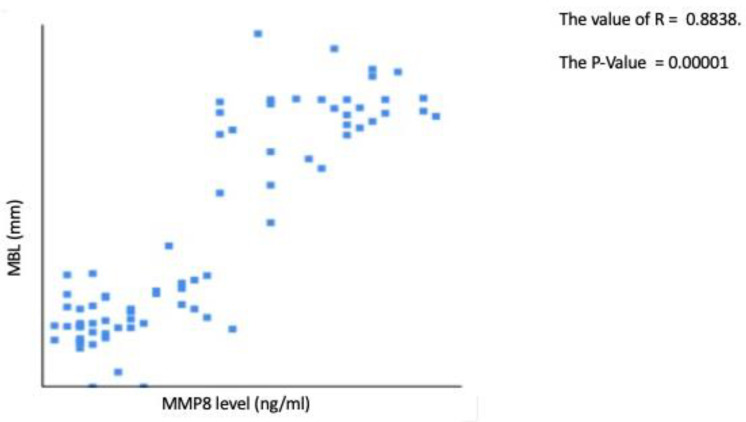
Pearson correlation between MBL and MMP-8 concentration at T24.

aMMP-8: Receiver operating curve analysis (Figure 3) for aMMP-8 levels indicated that the optimal cutoff value for categorizing implants as high BLT or low BLT was 15.6 ng/mL SD 3.2 ng/mL. Implants categorized as low BLT presented at T6 a mean aMMP-8 concentration of 12.6 ng/mL (SD 2.8), while around high BLT implants, the mean concentration of aMMP-8 was 26.9 ng/mL (SD 11.4). The difference was statistically significant (*p* < 0.001). At T24 around implants categorized as low BLT, the aMMP-8 mean level was 7.13 ng/mL (SD 2.1), while around implants categorized as high BLT, it was 22.45 ng/mL (SD 9.8). The difference was statistically significant (*p* < 0.001). Overall, 91% of implants with aMMP-8 levels < 15.6 ng/mL (SD 3.2) at T6 presented low levels of aMMP-8 at T24, while 96% of implants with aMMP8 high levels at T24 presented also aMMP-8 levels > 15.6 ng/mL (SD 3.2) at T6. In Figure 5, Figure 6 and Figure 7, the Pearson correlation at T6 and T24 between the MBL level and the MMP-8 concentration is reported. 

## 4. Discussion

Maintenance of peri-implant marginal bone level is a key criterion for implant success. Many thresholds of acceptable marginal bone loss are reported. The widely adopted thresholds are: 0.1–0.2 mm of bone loss per year [39] or loss of 2 mm [40] after the first year of loading. Other reported thresholds include: 2.5 mm bone loss after 5 years [41] and 1–1.5 mm [42] or 0.4 mm [43] from the time-point of loading. Although these bone loss thresholds provide easy clinical ‘cut-offs’, they do not predict future MBL. Since marginal bone remodeling is a dynamic process, the rate of MBL has been proposed as a better index of implant success than bone loss or bone level values [6,7]. The rate of MBL has been correlated with the time-duration (pre-, post-loading, and overall periods) [7]. A statistically significant correlation between high early MBL and worse marginal bone levels has also been documented [6]. In particular, a previous study by Galindo-Moreno et al. [6] dicated a threshold of MBL > 1.325 mm at 18 months, to distinguish implants with low and high BLT. 

The results of the present study show that high MBL rates at T24 were strongly associated with elevated rates between T0 and T6. Almost all the implants (96.1%) with MBL of >2 mm at 24 months had a high bone loss rate at 6 months. MBL rates are insignificant from implant placement to T0 in comparison with those between T0 and T6, and become almost stable between T6 and T24 (Figure 2). Based on the results obtained, it can be assumed that the optimal cutoff value for categorizing implants as high BLT or low BLT was 1.501 ± 0.33 mm, with a MBL rate > 0.38 mm/m ± 0.01 MBL at T0 and >0.0765 mm/m ± 0.02 at T6. Moreover, our findings suggest that MBL is more related to the prosthetic phase than to the post-surgical bone healing and remodeling process. These data are aligned with what was reported by Galindo-Moreno et al. [6], and indicate that the MBL rate immediately after restoration delivery may represent a clear risk indicator for implants to reach an MBL failure level over the medium or long term. An interesting result of the current study is related to MBL rate between implants with an LM vs. M collar surface, which at T0, T26, and T24 presented a mean MBL of 0.469 ± 0.2 mm vs. 0.732 ± 0.4 mm, 0.512 ± 0.3 mm vs. 1.424 ± 0.7 mm, and 0.63 ± 0.26 mm vs. 1.674 ± 0.9 mm, respectively. It is widely documented in the literature [44] that the laser-microtextured implant collar surface allows the physical attachment of connective tissues which present with a high mechanical stability with functional orientation of the connective fibers. Therefore, it is possible to speculate that around LM implants, the presence of an anatomical structure, likely of organized connective tissue, may protect the underlying bone after the implant functional loading.

As regards the variables taken into consideration, the results of the current study indicate that the implant collar surface (*p* < 0.001), time (*p* < 0.001), and smoking (*p* < 0.001) yielded significant effects on MBL progression. 

Determination of aMMP-8 levels in PISF has been shown to be useful for screening susceptible sites and patients, to differentiate the peri-implant sites, and to evaluate progression of bone loss in peri-implantitis [28,29,30,31,45]. The results of the current study show that the peri-implant marginal bone loss progression is statistically correlated with peri-implant sulcular fluid levels of metalloproteinase-8. Moreover, the initial high levels of marginal bone loss and metalloproteinase-8 can be considered as indicators of the subsequent progression of peri-implant MBL: implants with increased marginal bone loss rates and metalloproteinase-8 levels at 6 months after loading are likely to achieve additional marginal bone loss values. 

Although pathologically aMMP-8 elevated levels have been shown to be associated with to the early active phase of peri-implant diseases [45], as far as the authors are aware, no data are available in the literature on a possible correlation between the sulcular concentration of MMP-8 and the rates of marginal bone loss in implant sites. MMP-8 was also detected in the PISF of patients not affected by peri-implant mucositis or peri-implantitis, although the level of this marker was always lower than in the PISF collected from peri-implant mucositis/peri-implantitis patients [27,46]. Arakawa et al. [45] did not find the presence of MMP-8 in PISF collected from patients without clinical symptoms of inflammation around the implants, while Xu et al. [47] found that the concentration of MMP-8 in PISF in healthy implants is very low. Ma et al. [28] demonstrated that MMP-8 levels in PISF were associated with more bone loss around diseased implants. Lähteenmäki et al. [36] and Alassiri et al. [32] suggested a cut-off value < 20 ng/mL to differentiate peri-implantitis sites from clinically healthy sites. In the current study, the levels of the MMP-8 in PISF were detected at the restoration delivery (T0), and after 6/24 (T6/T24) months of functional loading. Receiver operating curve analysis (Figure 4) indicated that the optimal cutoff aMMP-8 concentration in PISF for categorizing implants as high BLT or low BLT was 15.6 ± 3.2 ng/mL at 6 months. Almost all of the implants (94%) with aMMP-8 levels > 15.6 ng/mL at 6 months had a high bone loss rate at 24 months. These data seem to suggest that the concentration of aMMP-8 has good reliability in identifying implants that could present a further MBL in the future long before X-ray evaluation. In a previous study (28), peri-implant MBL was graded vertically as being <1 mm, from 1 to 3 mm, or >3 mm. The aMMP-8 PISF concentration was significantly higher in the group with >3 mm of MBL (*p* = 0.049) than in those groups that had lost less bone. The Gi was slightly higher in the group with 1 to 3 mm of MBL than in the group with <1 mm of MBL, and it was highest in the group with >3 mm of MBL. However, none of these differences were significant (*p* > 0.05). In the present study, the mPI and PD findings do not correlate with the MBL level and aMMP-8 concentration, while a correlation was found with BoP. According to the collected data, PD does not seem to be reflected by the severity of peri-implant mucosal inflammation, as detected by the concentrations of aMMP-8 in PISF. 

The process of MBL still needs to be clarified; however, to date, there is more and more evidence in the literature that suggests that peri-implant MBL is generally an example of an imbalance immunological reaction due to non-optimal implant components, surgery, prosthodontics and/or compromised patient factors, refs. [4,48,49,50]. It seems to link to an adaptive response that could involve aseptic reactions typically limited to a few millimeters away from the interfaces, that are not always related to a disease state. In fact, most implant sites that exhibit initial crestal bone loss of <1 mm do not appear to be associated with soft tissue inflammation but have an apparently healthy peri-implant mucosa [51]. The hypothesis that MBL around implants is linked to a foreign body reaction was recently also supported by a transcriptome profiling study [52], indicating that the regulation of transcripts is related primarily to innate immune responses rather than to defense responses. Bacteria are not needed to trigger bone resorption around the dental implant [53], but whether bacteria will worsen the bone resorption or not is another issue. MMP-8, also known as “neutrophil collagenase”, is synthesized during the myelocyte stage of the development of the neutrophils, stored in the specific or secondary granules and released first upon activation of the cells [54]. MMPs are involved in bone resorption by removing organic bone matrix and generating collagen fragments that could activate osteoclasts [55]. Additionally, MMPs’ activity is thought to act over preosteoclast recruitment to the bone tissue and migration within the marrow to sites for osteoclast differentiation and bone resorption [56,57]. Collagenase-mediated tissue destruction has been suggested to have a role in foreign-body host response [54] and in active osteoclastic activity in peri-implant lesions [27,58]. In the MBL process, the induction of MMPs may in part relate by the production of debris from implants and, by means of a vicious circle, contribute to the further release of particles, which may in part be responsible for a further peri-implant bone resorption [54]. 

The major limitations of this study include its retrospective design, modest sample size, short observation periods and the limited number of potential predictors addressed. All the implants analyzed were placed by experts. Operator experience and competency are variables that can impact surgical and prosthetic accuracy [59]. The lack of cases treated by trainees in this population is a possible source of bias [59]. Many implant-related variables such as abutment height or soft-tissue thickness were not evaluated. 

## 5. Conclusions

Considering all these factors that could weaken the statistical robustness of the present study, it can be concluded that:

- Implants with increased MBL rates at 6 months after loading are likely to achieve additional MBL values.
- The initial high level of aMMP8 can be considered as indicators of the subsequent progression of peri-implant bone loss.
- MMP-8 could be used as biomarker for identifying implants and patients that could present an HBL.

## Figures and Tables

**Table 1:** Frequencies for each level of the categorical factors.

Factors	Level	*n*	%
Location	Right mandible	16	20
	Left mandible	22	27.5
	Right mandible	24	30
	Right maxilla	18	22.5
Collar surface	LM	41	51.2
	M	39	48.8
Gender	Female	52	65
	Male	38	35
Alcohol	No	72	90
	Yes	8	10
Smoking	No	60	75
	Yes	20	25
Plaque Index	0	3	3.7
	1	48	60
	2	24	30
	3	5	6.3
PD	No	11	13.7
	Yes	69	86.3
BoP	No	68	85
	At least 1 site	12	15

**Table 2:** Descriptive statistics for metric variables.

	Mean (Median)	Standard Error of the Mean	Range	
Age	54.6 (53)	0.42	29	74
Smoking	14.6 (11.5)	0.47	0	30
Implant diameter	3.95 (4)	0.02	3.8	4.6
Implant length	10.6	0.06	9.5	12
MBL (mm) at T0	0.29 (0.21)	0.018	0.2	0.6
MBL (mm) at T6	0.95 (0.53)	0.075	0.2	2.4
MBL (mm) at T24	1.4 (1.22)	0.10	0.2	3.2

**Table 3:** Mean rates, standard errors (), and median rates [] for factors explaining the variance in the dependent variables at the three measurements times (rates are expressed in mm/month).

		T0	T6	T24
BLT	Low High	0.041 (0.058 (0.03) 0.058 (0.03) [0.02]	0.097 (0.04) [0.02] 0.192 (0.07) [0.12]	0.014 (0.02) [0.01] 0.042 (0.02) [0.02]
Collar surface	LM M	0.066 (0.01) [0] 0.112 (0.04) [0.08]	0.082 (0.03) [0.05] 0.253 (0.18) [0.14]	0.044 (0.02) [0.02] 0.063 (0.03) [0.03]
PD	No Yes	0.031 (0.00) [0.00] 0.052 (0.00) [0.00]	0.058 (0.00) [0.02] 0.074 (0.01) [0.01]	0.037 (0.03) [0.02] 0.055 (0.019 [0.02]
BoP	No Yes *	0.011 (0.00) [0.00] 0.064 (0.02) [0.01]	0.097 (0.04) [0.02] 0.134 (0.03) [0.02]	0.073 (0.029 [0.03] 0.65 (0.03) [0.03]
Smoking *		0.094 (0.06) 0.00]	0.134 (0.03) [0.02]	0.65 (0.03) [0.03]
Gender	Female Male	0.038 (0.00) [0.00] 0.039 (0.00) [0.001]	0.065 (0.00) [0.01] 0.061 (0.01) [0.01]	0.029 (001) [0.01] 0.031 (0.00) [0.01]
Age *		0.066 (0.03)	0.021 (0.03)	0.54 (0.03)

* Pearson linear correlation.

**Figure A1:**
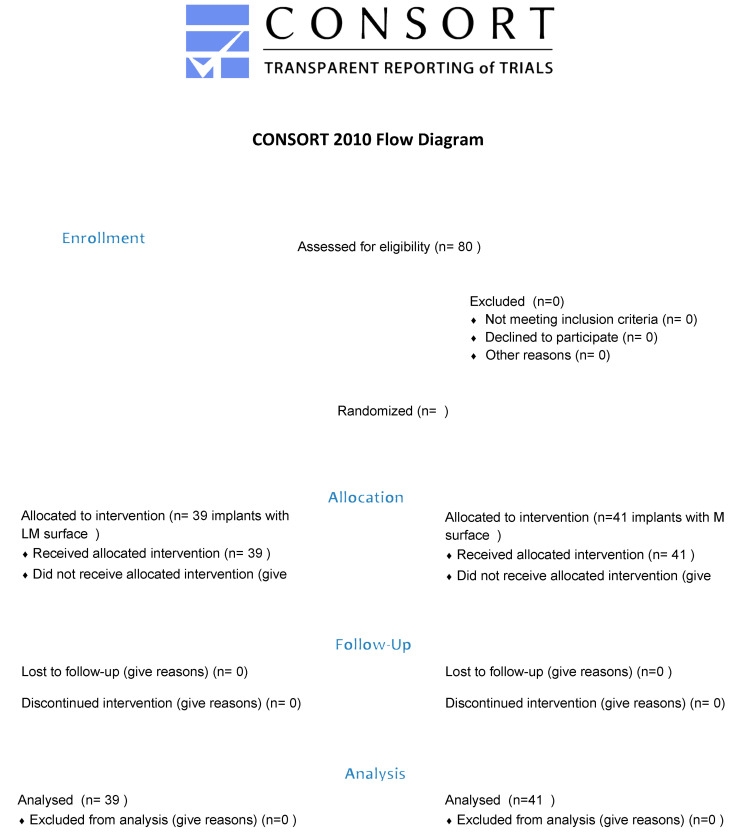
The CONSORT Flow Diagram.
